# Blockade of Kv1.3 Potassium Channel Inhibits Microglia-Mediated Neuroinflammation in Epilepsy

**DOI:** 10.3390/ijms232314693

**Published:** 2022-11-24

**Authors:** Xinyi Zhang, Peiyu Liang, Yahui Zhang, Yifan Wu, Yinghao Song, Xueyang Wang, Taoxiang Chen, Biwen Peng, Wanhong Liu, Jun Yin, Song Han, Xiaohua He

**Affiliations:** 1 Department of Pathophysiology, Taikang Medical School (School of Basic Medical Sciences), Wuhan University, Wuhan 430071, China; 2 Hubei Provincial Key Laboratory of Developmentally Originated Disease, Taikang Medical School (School of Basic Medical Sciences), Wuhan University, Wuhan 430071, China; 3 Department of Physiology, Taikang Medical School (School of Basic Medical Sciences), Wuhan University, Wuhan 430071, China; 4 Department of Immunology, Taikang Medical School (School of Basic Medical Sciences), Wuhan University, Wuhan 430071, China

**Keywords:** epilepsy, microglia, Kv1.3, inflammation, calcium, NF-κB signaling pathway

## Abstract

Epilepsy is a chronic neurological disorder whose pathophysiology relates to inflammation. The potassium channel Kv1.3 in microglia has been reported as a promising therapeutic target in neurological diseases in which neuroinflammation is involved, such as multiple sclerosis (MS), Alzheimer’s disease (AD), Parkinson’s disease (PD), and middle cerebral artery occlusion/reperfusion (MCAO/R). Currently, little is known about the relationship between Kv1.3 and epilepsy. In this study, we found that Kv1.3 was upregulated in microglia in the KA-induced mouse epilepsy model. Importantly, blocking Kv1.3 with its specific small-molecule blocker 5-(4-phenoxybutoxy)psoralen (PAP-1) reduced seizure severity, prolonged seizure latency, and decreased neuronal loss. Mechanistically, we further confirmed that blockade of Kv1.3 suppressed proinflammatory microglial activation and reduced proinflammatory cytokine production by inhibiting the Ca^2+^/NF-κB signaling pathway. These results shed light on the critical function of microglial Kv1.3 in epilepsy and provided a potential therapeutic target.

## 1. Introduction

Epilepsy is a chronic neurological disorder characterized by spontaneous recurrent seizures. Approximately 50 million people worldwide suffer from epilepsy [1,2], which seriously affects the quality of life of patients. Although several factors (including traumatic brain injury, gene mutations, and infections) have been identified as causes of epilepsy [3,4,5], approximately 60% of epileptic disorders have unknown etiology. At present, surgery and antiepileptic drugs can prevent 70% of patients from seizures [6]. However, due to the existence of drug resistance, there are still a considerable number of patients who cannot be effectively treated. Therefore, there is an urgent need to develop novel antiepileptic drugs.

One of the vital pathophysiological hallmarks of epilepsy is neuroinflammation, which predominantly involves activated microglia and astrocytes releasing many types of inflammatory mediators [7,8]. A previous study manifested that proinflammatory cytokines increased neuronal excitability and lowered seizure threshold [9]. The concentration of proinflammatory cytokines detected in cerebral spinal fluid (CSF) was higher in epileptic patients than in healthy volunteers, further implying that the activation of cytokine cascade and associated inflammatory signals was responsible for epileptogenesis [10]. Additionally, dysregulated neuroinflammatory signals contribute to seizure generation, specifically reflected in the brain discharge and neuronal damage [11]. It was reported that downregulated hippocampal expression of proinflammatory cytokines drastically reduced the frequency of spontaneous seizures and shortened the duration in KA-induced epilepsy mouse model [12]. Many clinical studies have also demonstrated that various anti-inflammatory drugs showed therapeutic effects on pharmacoresistant seizures [13,14,15]. Hence, currently inhibiting inflammation is regarded as a therapeutic approach for epilepsy [16].

Microglia, as the first line of defense in the central nervous system, are the main effectors of neuroinflammatory processes. Seizures cause microglia to proliferate and activate and release large amounts of inflammatory cytokines. The initially activated microglia are responsible for subsequently inducing epileptogenic reactive astrocytes [17]. At present, interventions in the inflammatory activation of microglia may offer potential targets for the treatment of epilepsy. It was reported that inhibiting proinflammatory microglia/macrophages responses by conditional knockout of ASK1 reduced seizure severity and histological damage [18].

The potassium channels have been reported to be the causes of several types of epilepsy and regarded as prominent targets for the treatment [4,19,20]. Most of the research on potassium channels and epilepsy has focused on neuronal excitability, independent of neuroinflammation. The potassium channel Kv1.3 has attracted extensive attention due to its immunomodulatory function. It was first described in human T cells [21], and then it was also identified in B lymphocytes, macrophages, and microglia [22,23,24]. Kv1.3 knockout reduced microglial proinflammatory response after lipopolysaccharide (LPS) injection. Recently, microglial Kv1.3 has been reported to be related to a variety of neurological diseases in which neuroinflammation is involved, such as Parkinson’s disease (PD), Alzheimer’s disease (AD), and middle cerebral artery occlusion/reperfusion (MCAO/R) [25,26,27,28]. The expression of Kv1.3 was increased in these diseases and has been suggested to act as a pharmacological target. However, it remains unclear whether the microglial Kv1.3 channel is involved in the process of epileptogenesis. Given the critical role of neuroinflammation in epilepsy, we hypothesized that blocking microglial Kv1.3 might mitigate the neuroinflammatory responses, thereby alleviating neuronal injury and seizures.

At present, kainic acid (KA) is considered as an agonist to induce experimental epilepsy in rodents. It has been widely used over the past few decades [29,30] due to its ability to reproduce highly similar neuropathological and electroencephalographic features observed in patients with temporal lobe epilepsy (TLE).

To investigate the function and related mechanism of microglia in epilepsy, a cell model is needed for in vitro studies. However, currently there is no suitable cell model that can reproduce the inflammatory environment of epilepsy. Hence, it is accepted that lipopolysaccharide (LPS)-stimulated microglia are used as an inflammatory model to explore the mechanism of epilepsy [31,32,33].

In this study, we focused on the role of microglial Kv1.3 in epilepsy. Our in vivo studies showed that microglial Kv1.3 was upregulated in KA-induced epileptic mice, and a treatment of 5-(4-phenoxybutoxy)psoralen (PAP-1), the blocker of Kv1.3 (EC_50_ = 2 nM), alleviated seizures. Then, PAP-1 was confirmed to mitigate epilepsy and inhibit the inflammatory activation of microglia in vivo and in vitro. Finally, we explored the underlying molecular mechanisms of Kv1.3 on modulating microglia-induced inflammation using an LPS-stimulated cell model. Mechanistically, PAP-1 inhibited the inflammatory activation of microglia and the release of inflammatory cytokines by inhibiting the Ca^2+^/NF-κB signaling pathway.

## 2. Results

### 2.1. Kv1.3 Potassium Channel in Microglia Is Upregulated in KA-Induced Epileptic Mice

Since Kv1.3 expression has been reported to increase in microglia in some neurological diseases such as AD and PD [25,27], we investigated whether the expression of Kv1.3 was changed in brains of KA-induced epileptic mice compared with the Ctrl group.

**Figure 1:**
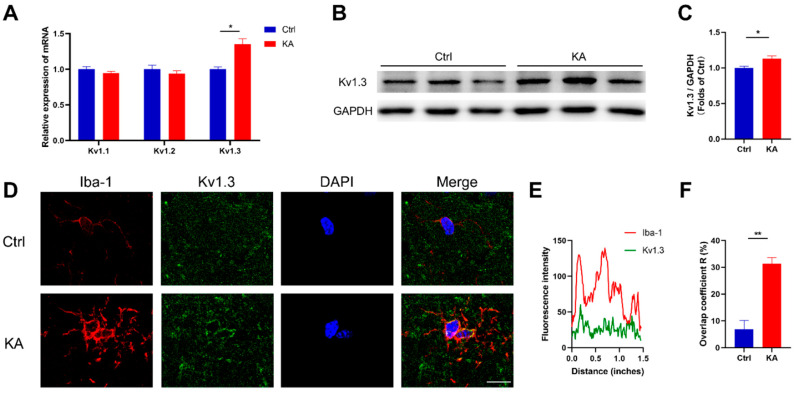
Kv1.3 was upregulated in microglia in KA-induced epileptic mice. (**A**) mRNA levels of potassium channels were detected in the brain of mice in Ctrl (n = 4 to 5) and KA (n = 9) groups; statistics by unpaired *t*-test. (**B**,**C**) Western blot analysis of Kv1.3 in the brain of mice in Ctrl and KA groups (n = 3); statistics by unpaired *t*-test. (**D**–**F**) Representative confocal images of Iba-1/Kv1.3/DAPI staining in the hippocampus of the two groups and colocalization analysis of Iba-1 and Kv1.3 (n = 3); statistics by unpaired *t*-test. Scale bar = 10 μm. Data are depicted as the mean ± SEM. * *p* < 0.05, ** *p* < 0.01.

We found that the mRNA level of Kv1.3 was increased significantly in the brains of KA-induced epileptic mice (*p* = 0.0156), while the mRNA levels of other potassium channels such as Kv1.1 (*p* = 0.2347) or Kv1.2 (*p* = 0.4045) were not changed (Figure 1A). Likewise, an increase of Kv1.3 protein level was found in the brains of KA mice (*p* = 0.0404, Figure 1B,C), suggesting that the Kv1.3 channel was involved in the process of epilepsy. Moreover, we explored the cellular localization of Kv1.3 and confirmed that this upregulation of Kv1.3 was localized to Iba-1-positive microglia (*p* = 0.0037, Figure 1D–F), indicating that Kv1.3 may play a crucial role in mediating microglial activation in KA-induced epileptic mice.

Considering that neuroinflammation is critical in epilepsy, these results suggested a potential role of microglial Kv1.3 in epileptic pathogenesis and progression.

### 2.2. Blockade of Kv1.3 Attenuates KA-Induced Epilepsy

**Figure 2:**
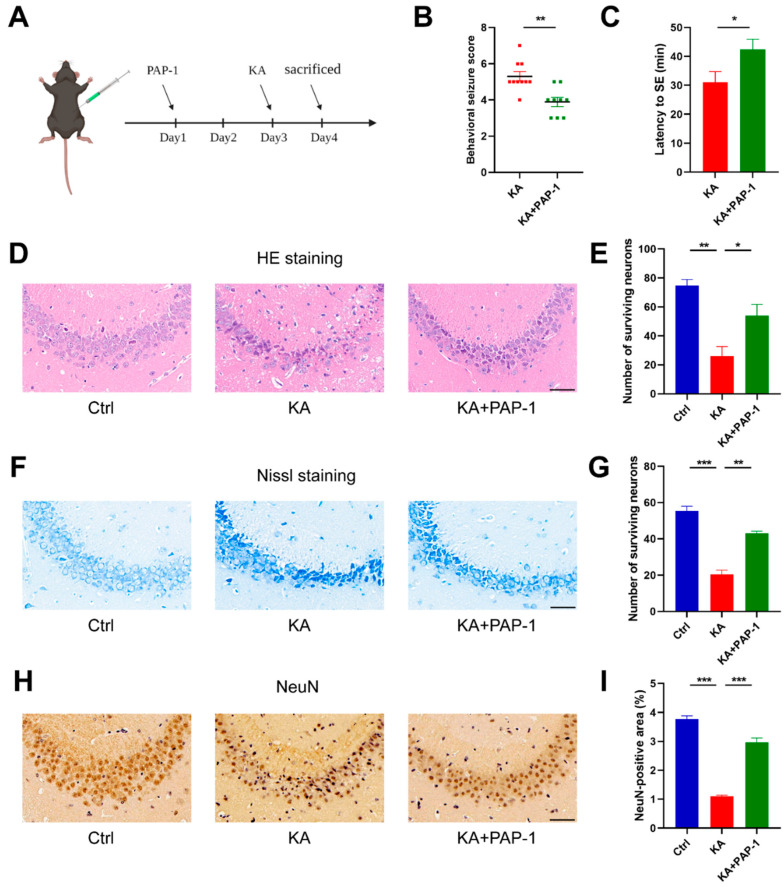
PAP-1 attenuated epileptic seizures and hippocampal neuronal damage in KA-induced mouse epilepsy model. (**A**) Flowchart of the experiment in vivo. (**B**) Seizure severity of mice after KA treatment in the presence (n = 9) or absence of PAP-1 (n = 10); statistics by Mann–Whitney test. (**C**) Seizure latency to SE; statistics by unpaired *t*-test. (**D**,**F**,**H**) Representative HE, Nissl, and NeuN staining of the hippocampal CA3 region. Scale bar: 50 μm. (**E**,**G**,**I**) Surviving neurons of each group were counted (n = 3); statistics by one-way ANOVA with Tukey’s multiple comparisons test; HE, F (2, 6) = 14.50, *p* = 0.0050; Nissl, F (2, 6) = 68.07, *p* < 0.0001; NeuN, F (2, 6) = 148.3, *p* < 0.0001. Data are depicted as the mean ± SEM. * *p* < 0.05, ** *p* < 0.01, *** *p* < 0.001.

To investigate the specific effect of microglial Kv1.3 on epilepsy, we assessed seizure severity and latency by observing neurobehaviors after treatment of PAP-1, a specific blocker of the Kv1.3 channel (Figure 2A). Then, we performed HE, Nissl, and NeuN staining to detect neuropathic changes after seizures.

The behaviors of the mice were observed and recorded for 2 h after KA injection. The results showed that mice of the KA+PAP-1 group had lower seizure severity (3.889 ± 0.2606 vs. 5.300 ± 0.2603, *p* = 0.0019) and longer seizure latency (42.38 ± 3.500 vs. 31.05 ± 3.730 min, *p* = 0.0418), compared with KA mice (Figure 2B,C). Next, we investigated neuronal loss in the CA3 region in the hippocampus, which is susceptible to KA. Compared with the Ctrl group, many neurons exhibited cytoplasmic shrinkage, triangulated pyknotic nuclei, and darker Nissl staining in the KA group (HE, *p* = 0.0041; Nissl, *p* < 0.0001). In contrast, most neurons showed standard cell shape in the KA+PAP-1 group, and the number of neurons with darker blue staining decreased (HE, *p* = 0.0489; Nissl, *p* = 0.0007, Figure 2D–G). In addition, KA-induced neuronal loss was relieved by PAP-1 treatment (KA vs. Ctrl, *p* < 0.0001; KA+PAP-1 vs. KA, *p* < 0.0001, Figure 2H,I).

Taken together, the Kv1.3 blocker PAP-1 dramatically ameliorated KA-induced hippocampal neuronal damage and epileptic seizures.

### 2.3. Blockade of Kv1.3 Inhibits the Activation of Microglia in KA-Induced Epileptic Mice

After we found that Kv1.3 blockade attenuated KA-induced epilepsy in mice, we explored how Kv1.3 blocker PAP-1 mitigated seizures and alleviated neuronal damage. Neuroinflammation promotes the occurrence of convulsions and neuronal damage after seizures, which is accompanied by the release of proinflammatory cytokines, such as IL-1β, IL-6, and TNF-α. These proinflammatory cytokines are released primarily by activated glia. Given the indispensable role of microglia in epilepsy, we examined the proliferation and inflammatory activation of microglia and related proinflammatory cytokine production with the treatment of Kv1.3 blocker PAP-1 in KA-induced epileptic mice.

**Figure 3:**
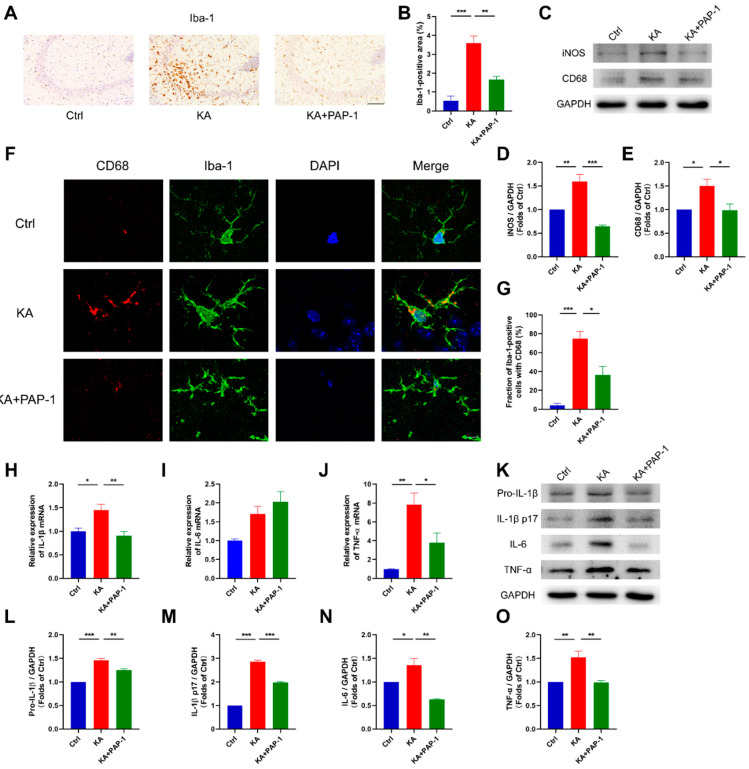
PAP-1 reduced microglial activation and proinflammatory cytokine production in vivo. (**A**,**B**) Iba-1 staining of the hippocampal CA3 region (n = 3); statistics by one-way ANOVA with Tukey’s multiple comparisons test, F (2, 6) = 31.65, *p* = 0.0006. Scale bar: 100 μm. (**C**–**E**) Representative Western blot images and statistical analysis of iNOS and CD68 in the brain (n = 3); statistics by one-way ANOVA with Tukey’s multiple comparisons test; iNOS, F (2, 6) = 28.18, *p* = 0.0009; CD68, F (2, 6) = 6.829, *p* = 0.0284. (**F**,**G**) Representative confocal images of CD68/Iba-1/DAPI staining in the hippocampus and colocalization analysis of CD68 and Iba-1 (n = 3); statistics by one-way ANOVA with Tukey’s multiple comparisons test, F (2, 6) = 25.06, *p* = 0.0012. Scale bar: 10 μm. (**H**–**J**) Gene expression of IL-1β, IL-6, and TNF-α in the brain (n = 4 to 7 per group); statistics by one-way ANOVA with Tukey’s multiple comparisons test; IL-1β, F (2, 14) = 7.849, *p* = 0.0052; IL-6, F (2, 14) = 4.443, *p* = 0.0321; TNF-α, F (2, 14) = 9.470, *p* = 0.0025. (**K**–**O**) The protein levels of Pro-IL-1β, IL-1β p17, IL-6, and TNF-α in the brain were detected by Western blot (n = 3); statistics by one-way ANOVA with Tukey’s multiple comparisons test; Pro-IL-1β, F (2, 6) = 88.03, *p* < 0.0001; IL-1β p17, F (2, 6) = 320.3, *p* < 0.0001; IL-6, F (2, 6) = 20.46, *p* = 0.0021; TNF-α, F (2, 6) = 14.52, *p* = 0.0050. Data are depicted as the mean ± SEM. * *p* < 0.05, ** *p* < 0.01, *** *p* < 0.001.

We first quantitate microgliosis with marker Iba-1. In the hippocampus, the expression of Iba-1 was upregulated in the KA group (*p* = 0.0005), and pretreatment with PAP-1 reduced KA-induced microgliosis (*p* = 0.0059, Figure 3A,B). Next, we examined the inflammatory activation of microglia. iNOS and CD68, the hallmarks of microglial inflammation, were both upregulated in the KA group (iNOS, *p* = 0.0084; CD68, *p* = 0.0448), while they were significantly downregulated in the KA+PAP-1 group (iNOS, *p* = 0.0007; CD68, *p* = 0.0405, Figure 3C–E). In addition, colocalization analysis showed that CD68 expression was increased in hippocampal microglia in KA mice (*p* = 0.0010), and the increase of CD68 protein level was inhibited by PAP-1 treatment (*p* = 0.0197, Figure 3F,G). These results suggested that blockade of Kv1.3 reduced microgliosis and prevented proinflammatory microglial activation in KA-induced epileptic mice. Then, we examined the expression of inflammatory mediators in vivo. The qPCR analysis revealed that blockade of Kv1.3 reduced KA-induced mRNA expression of the proinflammatory cytokines, except for IL-6 (IL-1β, *p* = 0.0058; IL-6, *p* = 0.5525; TNF-α, *p* = 0.0357, Figure 3H–J). Western blot analysis revealed an increase in protein levels of the proinflammatory cytokines IL-1β, IL-6, and TNF-α in KA-induced epileptic mice (Pro-IL-1β, *p* < 0.0001; IL-1β p17, *p* < 0.0001; IL-6, *p* = 0.0469; TNF-α, *p* = 0.0084), while this effect was inhibited by PAP-1 treatment (Pro-IL-1β, *p* = 0.0022; IL-1β p17, *p* < 0.0001; IL-6, *p* = 0.0017; TNF-α, *p* = 0.0080, Figure 3K–O).

These findings demonstrated that blockade of Kv1.3 dramatically reduced microglial activation and proinflammatory cytokine production in vivo and attenuated epilepsy as a consequence.

### 2.4. Blockade of Kv1.3 Suppresses Proinflammatory Activation of Microglia In Vitro

To further verify the anti-inflammatory effect of Kv1.3 blockade on microglia, we cultured BV2 microglia and primary microglia with LPS stimulation in vitro. We first examined the change of Kv1.3 expression in LPS-stimulated microglia and then investigated microglial activation and proinflammatory cytokine production to assess the effects of Kv1.3 blockade on reducing microglial inflammation.

**Figure 4:**
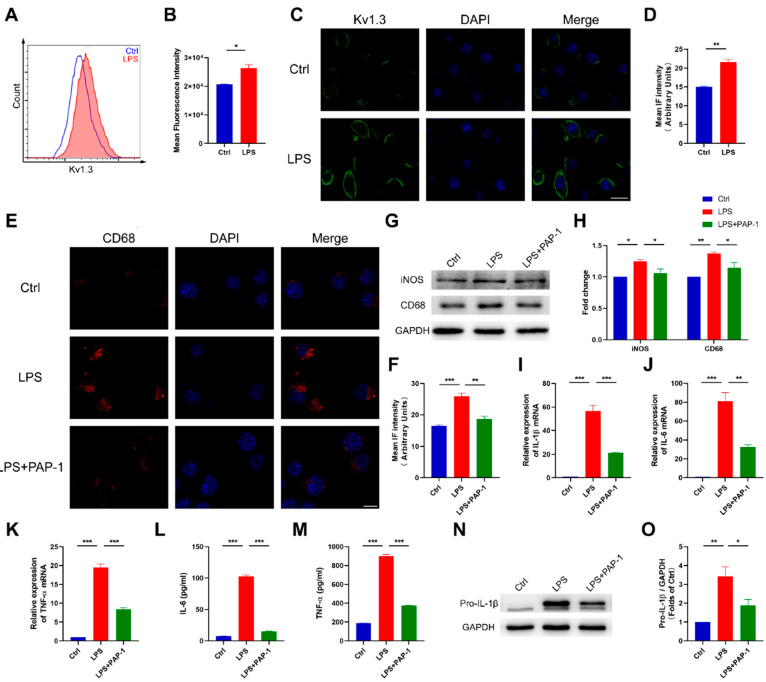
PAP-1 reduced microglial activation and proinflammatory cytokine production in vitro. (**A**,**B**) Flow cytometric analysis of the surface expression of Kv1.3 in BV2 microglia (n = 3); statistics by unpaired *t*-test with Welch’s correction. (**C**,**D**) Immunofluorescence of LPS-induced Kv1.3 protein expression in BV2 microglia (n = 3); statistics by unpaired *t*-test. Scale bar: 20 μm. (**E**,**F**) Representative confocal images showing CD68 expression after treatment in BV2 microglia and statistical analysis (n = 3); statistics by one-way ANOVA with Tukey’s multiple comparisons test, F (2, 6) = 37.55, *p* = 0.0004. Scale bar: 10 μm. (**G**, **H**) Cell lysates were immunoblotted with iNOS and CD68 antibodies, and the results were summarized (n = 3); statistics by one-way ANOVA with Tukey’s multiple comparisons test; iNOS, F (2, 6) = 8.702, *p* = 0.0168; CD68, F (2, 6) = 13.01, *p* = 0.0066. (**I**–**K**) Gene expression of IL-1β, IL-6, and TNF-α in BV2 microglia after treatment (n = 3); statistics by one-way ANOVA with Tukey’s multiple comparisons test; IL-1β, F (2, 6) = 109.2, *p* < 0.0001; IL-6, F (2, 6) = 53.60, *p* = 0.0001; TNF-α, F (2, 6) = 258.6, *p* < 0.0001. (**L**,**M**) IL-6 and TNF-α in the supernatants of BV2 microglia were measured using ELISA (n = 3); statistics by one-way ANOVA with Tukey’s multiple comparisons test; IL-6, F (2, 6) = 50.41, *p* = 0.0002; TNF-α, F (2, 6) = 45.93, *p* = 0.0002. (**N**,**O**) Cell lysates were immunoblotted with IL-1β antibody, and the results were summarized (n = 3); statistics by one-way ANOVA with Tukey’s multiple comparisons test, F (2, 6) = 34.35, *p* = 0.0005. Data are depicted as the mean ± SEM. * *p* < 0.05, ** *p* < 0.01, *** *p* < 0.001.

Using a FITC-labeled Kv1.3 antibody against an extracellular epitope, we found that LPS increased the surface expression of Kv1.3 in BV2 microglia by Flow cytometry (26,363 ± 1175 vs. 20,609 ± 138.6, *p* = 0.0377, Figure 4A,B). Furthermore, the Kv1.3 protein level also increased, as revealed by Immunofluorescence (*p* = 0.0015, Figure 4C,D). In primary microglia, a similar upregulation was shown (Figure S1A,B).

To further confirm the effect of Kv1.3 on modulating proinflammatory activation of microglia, we pretreated microglia with 10 μM PAP-1 before LPS stimulation in vitro and examined inflammatory activation and release of inflammatory cytokines. In the LPS stimulation group, the expression of CD68 was increased (*p* = 0.0004), and PAP-1 treatment inhibited this increase of CD68 protein level in BV2 microglia (*p* = 0.0017, Figure 4E,F). Likewise, PAP-1 reduced the protein levels of iNOS and CD68 in BV2 microglia induced by LPS (iNOS, *p* = 0.0495; CD68, *p* = 0.0480, Figure 4G,H). These results suggested that blockade of Kv1.3 prevented proinflammatory microglial activation after LPS stimulation. Next, we examined the production of proinflammatory cytokines. Our results showed that PAP-1 significantly inhibited the increase of proinflammatory cytokines IL-1β, IL-6, and TNF-α in the mRNA level in BV2 microglia stimulated by LPS (IL-1β, *p* = 0.0002; IL-6, *p* = 0.0020; TNF-α, *p* < 0.0001, Figure 4I–K). As expected, PAP-1 treatment significantly reduced LPS-induced protein expression of proinflammatory cytokines, including IL-1β (*p* = 0.0064), IL-6 (*p* = 0.0043), and TNF-α (*p* = 0.0306, Figure 4L–O). Furthermore, PAP-1 treatment also reduced LPS-induced microglial activation and proinflammatory cytokine production in primary microglia (Figure S2).

These findings suggested that blockade of Kv1.3 directly reduced microglial activation and proinflammatory cytokine production in vitro.

### 2.5. Blockade of Kv1.3 Attenuates Microglial Activation through the Ca^2+^/NF-κB Signaling Pathway

After we found that blockade of Kv1.3 could suppress proinflammatory microglia in vivo and in vitro, we then investigated the underlying molecular mechanism of the anti-inflammatory effect of Kv1.3 blocker PAP-1 in epilepsy. Considering that the NF-κB signaling pathway is associated strongly with inflammation, we examined whether it is upregulated in epilepsy and verified the inhibitory effect of PAP-1 on it.

**Figure 5:**
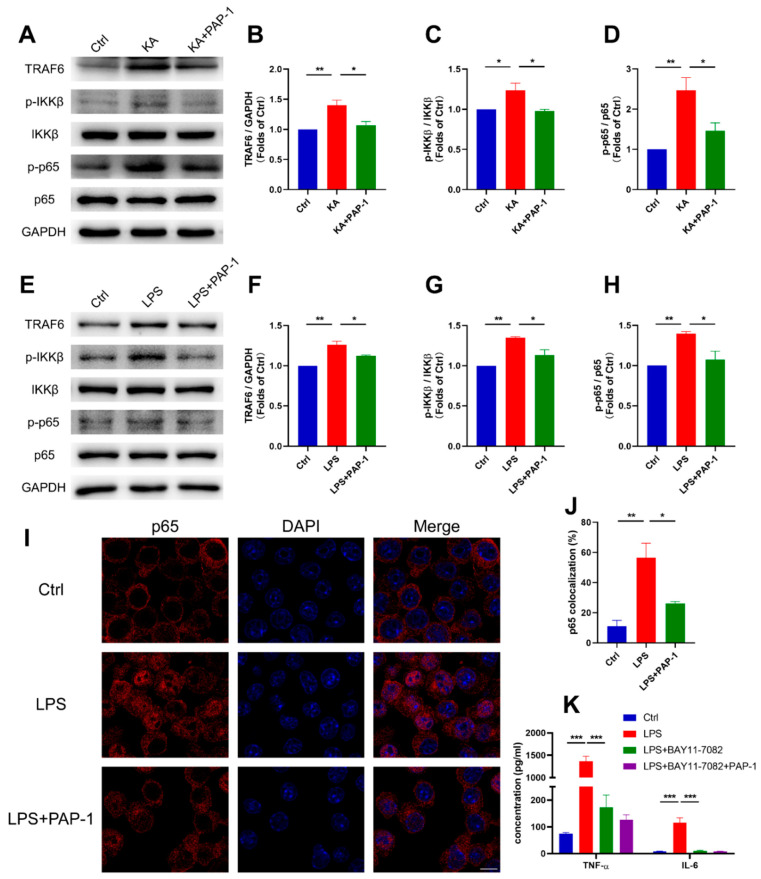
Kv1.3 blockade inhibited KA/LPS-induced microglial activation through NF-κB signaling pathway. (**A**–**D**) Western blot analysis of TRAF6, p-IKKβ, IKKβ, p-p65, and p65 in the brain (n = 3); statistics by one-way ANOVA with Tukey’s multiple comparisons test; TRAF6, F (2, 6) = 13.87, *p* = 0.0056; p-IKKβ/IKKβ, F (2, 6) = 7.799, *p* = 0.0214; p-P65/P65, F (2, 6) = 12.54, *p* = 0.0072. (**E**–**H**) Western blot analysis of TRAF6, p-IKKβ, IKKβ, p-p65, and p65 in BV2 microglia (n = 3); statistics by one-way ANOVA with Tukey’s multiple comparisons test; TRAF6, F (2, 6) = 24.23, *p* = 0.0013; p-IKKβ/IKKβ, F (2, 6) = 20.38, *p* = 0.0021; p-P65/P65, F (2, 6) = 11.63, *p* = 0.0086. (**I**,**J**) The nuclear translocation analysis of p65 in BV2 microglia (n = 3); statistics by one-way ANOVA with Tukey’s multiple comparisons test, F (2, 6) = 15.02, *p* = 0.0046. Scale bar: 5 μm. (**K**) IL-6 and TNF-α in the supernatants of BV2 microglia were measured using ELISA (n = 3); statistics by one-way ANOVA with Tukey’s multiple comparisons test; IL-6, F (3, 8) = 34.01, *p* < 0.0001; TNF-α, F (3, 8) = 105.0, *p* < 0.0001. Data are depicted as the mean ± SEM. * *p* < 0.05, ** *p* < 0.01, *** *p* < 0.001.

We found that the NF-κB pathway was activated by upregulating the expression of TRAF6, p-IKKβ, and p-p65 in both the KA-induced epileptic mice (*p* = 0.0063; *p* = 0.0395; *p* = 0.0065, respectively, Figure 5A–D) and LPS-induced inflammatory BV2 microglia (*p* = 0.0011, *p* = 0.0018, *p* = 0.0094, respectively, Figure 5E–H). Notably, blocking Kv1.3 with PAP-1 rescued the KA/LPS-induced increase of TRAF6 protein level and the ratio of p-IKKβ/IKKβ and p-p65/p65 (KA+PAP-1, *p* = 0.0156, *p* = 0.0277, *p* = 0.0350, respectively; LPS+PAP-1, *p* = 0.0253, *p* = 0.0189, *p* = 0.0240, respectively, Figure 5A–H). Then we found that Kv1.3 blockade suppressed p65 nuclear translocation in LPS-stimulated BV2 microglia (*p* = 0.0267, Figure 5I,J). Furthermore, to further determine that PAP-1 inhibited inflammation through the NF-κB signaling pathway, we treated BV2 microglia with 5 μM BAY11-7082 (an inhibitor of the NF-κB signaling pathway) before LPS stimulation and found that BAY11-7082 attenuated the release of IL-6 (*p* = 0.0002) and TNF-α (*p* < 0.0001, Figure 5K). In addition, PAP-1 did not show a further influence in the presence of BAY11-7082 (IL-6, *p* = 0.9985; TNF-α, *p* = 0.9434, Figure 5K).

Collectively, these results suggested that Kv1.3 blockade may suppress microglial activation and proinflammatory cytokine production by inhibiting NF-κB pathway.

Recent studies have suggested that blocking Kv1.3 disrupted Ca^2+^ influx [34], and calcium signaling participated in the inflammation of microglia as a critical secondary messenger [35]. Thus, we speculated that calcium signaling might be involved in inhibiting microglial activation and proinflammatory cytokine production induced by PAP-1 and acted upstream of NF-κB signaling pathway. We measured the concentration of intracellular calcium in BV2 microglia treated with PAP-1 and LPS and then detected the expression of the NF-κB signaling pathway and proinflammatory cytokines after chelating cytosolic calcium.

**Figure 6:**
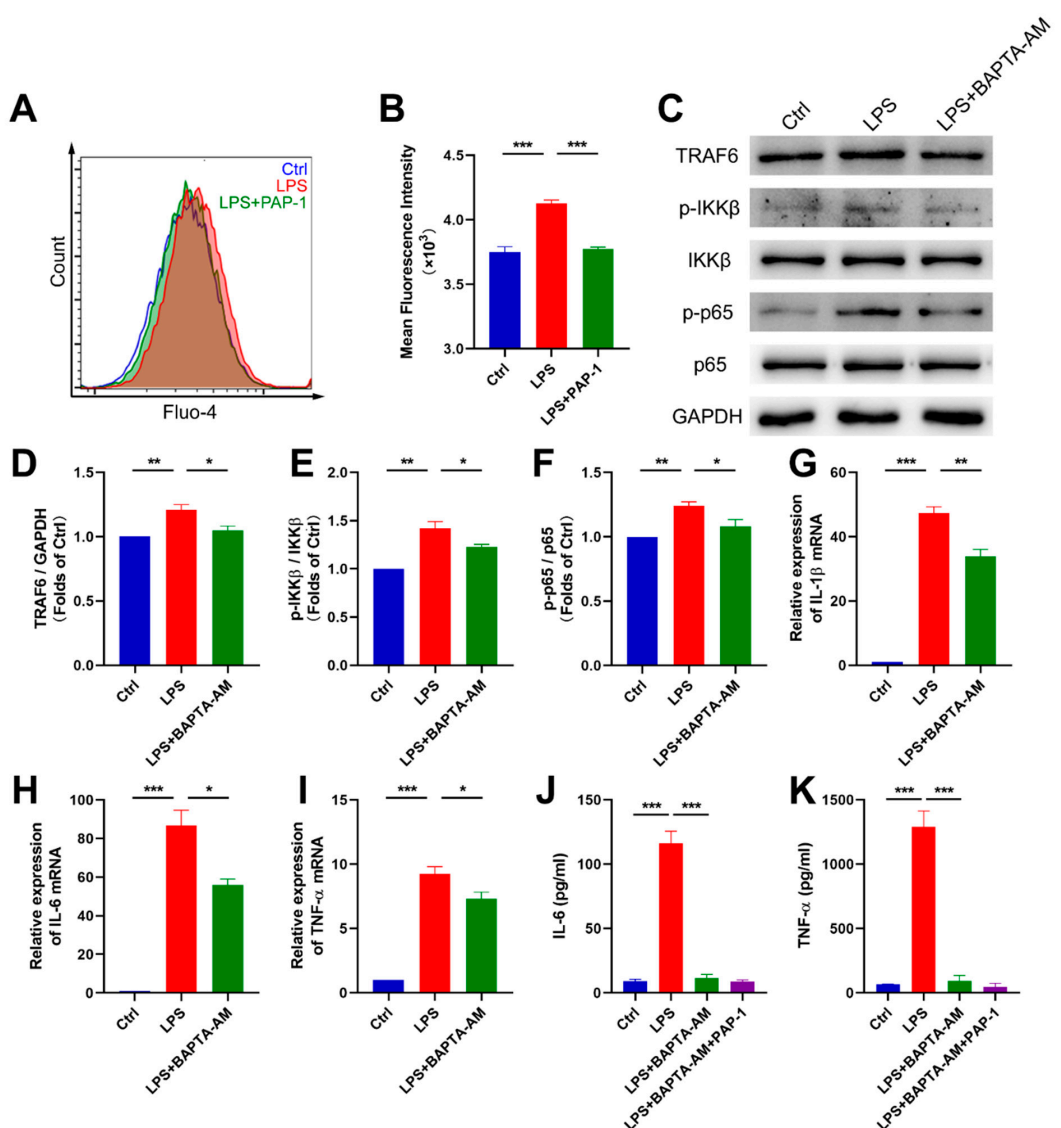
Kv1.3 blockade downregulated the NF-κB pathway via Ca^2+^ signaling. (**A**,**B**) Flow cytometric analysis of the intracellular Ca^2+^ level in BV2 microglia (n = 3); statistics by one-way ANOVA with Tukey’s multiple comparisons test; F (2, 6) = 49.05, *p* = 0.0002. (**C**–**F**) Representative Western blot images and statistical analysis of the expression levels of NF-κB signaling pathway in BV2 microglia (n = 3); statistics by one-way ANOVA with Tukey’s multiple comparisons test; TRAF6, F (2, 6) = 12.87, *p* = 0.0068; p-IKKβ/IKKβ, F (2, 6) = 22.83, *p* = 0.0016; p-P65/P65, F (2, 6) = 12.08, *p* = 0.0079. (**G**–**I**) Gene expression of IL-1β, IL-6 and TNF-α in BV2 microglia (n = 3); statistics by one-way ANOVA with Tukey’s multiple comparisons test; IL-1β, F (2, 6) = 193.5, *p* < 0.0001; IL-6, F (2, 6) = 77.72, *p* < 0.0001; TNF-α, F (2, 6) = 96.72, *p* < 0.0001. (**J**,**K**) IL-6 and TNF-α in the supernatants of BV2 microglia were measured using ELISA (n = 3); statistics by one-way ANOVA with Tukey’s multiple comparisons test; IL-6, F (3, 8) = 111.1, *p* < 0.0001; TNF-α, F (3, 8) = 85.29, *p* < 0.0001. Data are depicted as the mean ± SEM. * *p* < 0.05, ** *p* < 0.01, *** *p* < 0.001.

We first determined the cytosolic Ca^2+^ level of BV2 microglia by Flow cytometry using the fluorescence dye indicator Fluo-4 AM. The results showed that LPS led to an increase in intracellular Ca^2+^ concentration (4125 ± 28.29 vs. 3750 ± 41.03, *p* = 0.0003), and this increase was suppressed by PAP-1 treatment (3774 ± 14.47 vs. 4125 ± 28.29, *p* = 0.0004, Figure 6A,B). Then, to further investigate the role of calcium in inflammation, we incubated BV2 microglia with BAPTA-AM (10 μM) for 30 min, aiming to chelate cytosolic calcium. Western blot showed that Ca^2+^ chelation significantly inhibited the upregulation of the protein level of TRAF6 (*p* = 0.0235) and the increase of the ratio of *p*-IKKβ/IKKβ and p-p65/p65 induced by LPS (*p* = 0.0466; *p* = 0.0430, respectively, Figure 6C–F), which indicated that Ca^2+^ chelation could inhibit the activation of NF-κB signaling pathway. Last, we evaluated the expression of proinflammatory cytokines. Ca^2+^ chelation inhibited the upregulation of the mRNA level of proinflammatory cytokines induced by LPS (IL-1β, *p* = 0.0034; IL-6, *p* = 0.0106; TNF-α, *p* = 0.0490, Figure 6G–I). Likewise, Ca^2+^ chelation decreased the concentration of IL-6 (*p* < 0.0001) and TNF-α (*p* < 0.0001) after LPS stimulation, while PAP-1 did not show a further inhibition in the presence of BAPTA-AM (*p* = 0.9811; *p* = 0.9596, respectively, Figure 6J,K).

Overall, Kv1.3 blocker PAP-1 inhibited microglial activation and reduced proinflammatory cytokine production via the Ca^2+^/NF-κB signaling pathway.

## 3. Discussion

Epilepsy is a complex multifactorial disease, and it is difficult to perform an effective treatment because of pharmacoresistance [6,36,37]. In recent years, the research of epilepsy has been focused on discovering new therapeutic targets. Here, we tried to illustrate the role of the microglial Kv1.3 channel in epilepsy. We demonstrated that microglial Kv1.3 was upregulated in KA-induced epileptic mice, and pharmacological blockade of the Kv1.3 channel inhibited the neuroinflammation and seizures via the Ca^2+^/NF-κB signaling pathway. Our results indicated that Kv1.3 played an essential role in microglial activation during epilepsy.

In recent studies, the potassium channels have attracted more attention as causes of epilepsy [19,20,38]. The conclusion that mutations of K^+^ channels can result in severe epilepsy is further supported by evidence from clinical research. Pathogenic mutations of Kv2.1 and Kv3.2 channels are accepted to be linked with epileptic encephalopathies, the rare form of epilepsy [39,40]. Kv7.3 channel mutations have been implicated in resulting in BFNE (benign familial neonatal epilepsy), which is characterized by developmental delay and intellectual disability [41]. It has been reported that mutations of Kv1.1 and Kv1.2 channels can lead to episodic ataxia with generalized/focal seizures [42,43]. However, little is known about the association between Kv1.3 and epilepsy. In this study, we found that microglial Kv1.3 was upregulated in KA-induced epileptic mice, suggesting a possible role of Kv1.3 in epileptic pathogenesis and progression. Therefore, we used a small-molecule inhibitor PAP-1, which has been widely used as a specific Kv1.3 blocker in diseases, to investigate the role of Kv1.3 [25,27,28]. Our results showed that PAP-1 attenuated KA-induced epilepsy. However, the role of the Kv1.3 channel in epilepsy has not been reported in any genetic mouse models of epilepsy, and we believed that Kv1.3 might be involved in those models and should be further investigated in future.

An earlier study reported that Kv1.3 blocker Psora-4 could not obviously affect the action potential (AP) properties (including AP peak value, AHP, and AP frequency) of neurons compared with the control [44]. Kv1.3 immunoreactivity in astrocytes was reported to be unaltered after seizures in gerbils [45]. Therefore, we focused on the function of Kv1.3 in microglia. Kv1.3 has been found to contribute to the migration of the BV2 cell line and primary microglia [46]. Moreover, the Kv1.3 channel in LPS-activated microglia contributed strongly to postnatal hippocampal neuronal death [47]. Notably, much effort has focused on inflammation mediated by Kv1.3. Following differentiation with LPS, microglia exhibited high Kv1.3 current density, and the expression of Kv1.3 was upregulated [48]. Moreover, blockade of the microglial Kv1.3 channel reduced the release of IL-6 from brain slices of mice [49]. Similarly, we showed that PAP-1 reduced microglial activation and proinflammatory cytokine production, such as IL-1β, IL-6, and TNF-α. In our work, the uniqueness of the mRNA expression of IL-6 in the brain might be related to the diversity of cell types and complex transcription mechanism. The importance of microglial Kv1.3 has gained more attention in multiple neurological diseases in which neuroinflammation is involved. PD is characterized mainly by the loss of dopaminergic neurons in the substantia nigra pars compacta (SNpc) region of the brain. Chronic administration of PAP-1 protected multiple animal models of PD from the loss of dopaminergic neurons and reduced neuroinflammation, resulting in lower motor deficits [25]. Likewise, PAP-1 attenuated AβO-induced microglial activation and microglial neurotoxicity. Blockade of the Kv1.3 channel had a neuroprotective effect on Alzheimer’s transgenic model via inhibiting microglial activation and enhancing the microglial amyloid-β clearance capacity, thereby improving behavioral deficits [27]. In addition, treatment with PAP-1 intraperitoneally polarized microglia towards the M2 phenotype and inhibited NLRP3 inflammasome activation in ischemic stroke [28]. Given the critical role of inflammation in epilepsy, we hypothesized that blocking Kv1.3 might attenuate KA-induced epilepsy. As expected, we found that Kv1.3 blockade with PAP-1 reduced seizure severity, extended seizure latency, and relieved neuronal damage. Furthermore, PAP-1 inhibited microglial activation and proinflammatory cytokine production in KA-induced epileptic mouse models. Activated neuroinflammatory microglia induced A1 neurotoxic astrocytes through secreting IL-1α, TNF, and C1q [50]. It was reported that A1 astrocytes promoted the progression of epilepsy [51]. Whether blockade of Kv1.3 regulated the phenotypic transition of astrocytes via microglia is an interesting research direction.

NF-κB signaling pathway is one of the classic pathways associated with inflammation. Nuclear Factor Kappa B (NF-κB) is a widely expressed transcription factor. Evidence suggests that NF-κB participates in multiple activities, such as immune responses, DNA transcription, and cancer [52,53,54]. The activation of the NF-κB signaling pathway induced by LPS involves several steps. Upon stimulation, TRAF6 is upregulated as an adaptor protein of TLR4/MyD88 and leads to the phosphorylation of IKK, degradation of IκB, and release of p65. The released p65 translocates to the nucleus and binds to specific sequences, resulting in numerous proinflammatory cytokines production [55]. Strong evidence suggests that NF-κB may play a key role in modulating seizure susceptibility via inflammation. The inactive NF-κB signaling pathway induced by the kappa opioid receptor (KOR) participated in suppressing neuronal injury and regulating microglial M2 polarization in epileptic rats [31]. Likewise, our laboratory also verified that sitagliptin (a DPP4 inhibitor) reduced the KA-induced activation of the NF-κB signaling pathway and suppressed the inflammatory response mediated by microglia in epilepsy [56]. Here, we found that PAP-1 inhibited the NF-κB signaling pathway, resulting in attenuation of both microglial activation and epilepsy severity.

In this work, we demonstrated that Kv1.3, playing an essential role in neuroinflammation, was upregulated in KA-induced mouse epilepsy models. Noteworthy, the blockade of Kv1.3 attenuated KA-induced epilepsy and inhibited microglial activation and the release of proinflammatory cytokines (such as IL-1β, IL-6, and TNF-α) through the Ca^2+^/NF-κB signaling pathway. We believe our findings provided a novel strategy for the potential therapy of pharmacoresistant epilepsy. Although we found that Ca^2+^ was involved in the downregulation of the NF-κB signaling pathway, further studies are still required to fully understand the interaction between Ca^2+^ and NF-κB signaling pathway in microglia.

## 4. Materials and Methods

### 4.1. Mice

Eight-week-old male C57BL/6J mice (20 ± 2 g body weight) were purchased from Wuhan University Center for Animal Experiment/ABSL-3 Laboratory. The animals were housed at 20 ± 2 °C with 60 ± 5% humidity and a 12 h light/12 h dark cycle. For the experiment duration, all mice had ad libitum access to standard mouse chow and water. All protocols involving mouse models were approved by the Institutional Animal Care and Use Committee of Wuhan University.

### 4.2. Mouse Model and Drug Administration

On the third day of PAP-1 (MedChemExpress, New Jersey, NJ, USA) or saline treatment, the seizure model was induced by intraperitoneal (i.p.) injection of KA (Sigma, St. Louis, MO, USA). Mice were randomly divided into three groups and were treated as follows: mice in KA + PAP-1 group (n = 9) received PAP-1 (20 mg/kg/d i.p.) for 3 days before KA injection (30 mg/kg/d i.p.), the KA group (n = 10) received an equal volume of saline for 3 days before KA injection (30 mg/kg/d i.p.), and mice in the Ctrl group (n = 8) received an equal volume of saline for 3 days.

The typical behavioral observation was performed for 2 h after KA administration. Over a 2 h time course, seizure severity was scored based on a modified Racine method [57]: (0) normal behavior, (1) chewing and drooling, (2) head nodding, (3) unilateral forelimb clonus, (4) bilateral forelimb clonus, (5) forelimb or hindlimb clonus with falling, (6) running or jumping seizure, and (7) tonic hindlimb extension.

### 4.3. Tissue Collection

Twenty-four hours after KA injection, the mice were anesthetized using isoflurane and then intracardially perfused with saline. The mouse brain was rapidly removed and cut sagittally; one half was stored at −80 °C, and the other half was fixed in 4% paraformaldehyde and processed for paraffin embedding or frozen sections.

### 4.4. Microglia Culture and Drug Treatment

As previously described, primary microglia were separated from primary mixed glial cultures prepared from newborn C57BL/6J mice [58]. The BV2 microglia were purchased from the China Center for Type Culture Collection (Wuhan, China). Both BV2 cells and primary microglia were cultured in a humidified 5% CO_2_ incubator at 37 °C. Primary microglia were cultured in DMEM-F12 media (Gibco, Carlsbad, CA, USA) supplemented with 10% fetal bovine serum (Gibco, Melbourne, Australia) and a penicillin–streptomycin solution (Biosharp, Hefei, China). BV2 microglia were cultured in DMEM media (Gibco, Carlsbad, CA, USA) supplemented with 10% fetal bovine serum (BI, Herzliya, Israel) and a penicillin–streptomycin solution (Biosharp, Hefei, China).

Primary microglia were pretreated with 10 μM PAP-1 for 1 h and then stimulated with 100 ng/mL LPS (Sigma, St. Louis, MO, USA) for 12 h. BV2 microglia were pretreated with 10 μM PAP-1 for 1 h and then stimulated with 1 μg/mL LPS for 12 h. Both cell supernatants and lysates were collected. LPS stimulation was extended to 24 h for ELISA. To observe p65 nuclear translocation after LPS stimulation, cells were fixed with 4% paraformaldehyde 45 min after exposure to LPS.

### 4.5. Nissl and HE Staining

Paraffin sections were dewaxed with xylene and then rehydrated in an ethanol gradient. For Nissl staining, paraffin sections were stained with a 1% toluidine blue solution (Boster Biotech, Wuhan, China). For HE staining, paraffin sections were immersed in hematoxylin solution for 3 min, soaked in a hydrochloric acid alcohol solution for 5 s, and soaked in eosin solution for 2 min.

### 4.6. Immunohistochemistry

Paraffin sections were dewaxed with xylene and then rehydrated in an ethanol gradient. After blocking with 10% goat serum, the sections were incubated overnight with Iba-1 (1:2000, Abcam, Cambridge, UK) and NeuN (1:400, Proteintech, Wuhan, China) antibodies. On the second day, the sections were incubated with a horseradish peroxidase (HRP)-conjugated anti-rabbit antibody (1:200, Proteintech, Wuhan, China) for 1 h at room temperature and developed with DAB peroxidase substrate (Beyotime Biotechnology, Shanghai, China). Finally, we recorded digital images with a light microscope (Olympus, Hamburg, Germany).

### 4.7. Immunofluorescence

Frozen sections were warmed from −80 °C to room temperature. Cell samples did not require special handling. After being fixed with methanol, permeabilized with 0.5% Triton X-100, and blocked with 10% goat serum, the sections were incubated at 4 °C overnight with primary antibodies against the following proteins: Iba-1 (1:400, Abcam, Cambridge, UK), Kv1.3 (1:100, Alomone Labs, Jerusalem, Israel), CD68 (1:100, Abcam, Cambridge, UK), and NF-κB p65 (1:400, Cell Signaling Technology, Danvers, MA, USA), and followed by the respective fluorescein-labeled secondary antibody (Abbkine, Wuhan, China). Then, nuclei were stained with 4′,6-diamidino-2-phenylindole (DAPI; 1:100,000, Sigma, St. Louis, MO, USA). The samples were observed with a Leica-LCS-SP8-STED confocal laser-scanning microscope (Leica Microsystems, Wetzlar, Germany).

### 4.8. Quantitative PCR

Total RNA from the mouse brain or microglia was extracted using TRIzol reagent (Invitrogen, Carlsbad, CA, USA). Then, reverse transcription was performed using the HiScript^®^III RT SuperMix for qPCR (+gDNA wiper) (Vazyme, Nanjing, China) according to the manufacturer’s protocol. Quantitative PCR (qPCR) was performed with ChamQ Universal SYBR qPCR Master Mix (Vazyme, Nanjing, China). The expression of target genes was normalized to GAPDH. The 2^−ΔΔCt^ relative quantification method was used to calculate the target genes’ expression. A complete list of primer sequences is provided in Table 1.

### 4.9. Western Blot

Tissues and cells were lysed on ice using radioimmunoprecipitation assay (RIPA; Biosharp, Hefei, China) buffer supplemented with phenylmethanesulfonyl fluoride (PMSF; Biosharp, Hefei, China), protease inhibitors (TargetMol, Boston, MA, USA), and phosphatase inhibitors (TargetMol, Boston, MA, USA). The total protein concentration was quantified using a BCA protein assay (Beyotime Biotechnology, Shanghai, China). The detailed western blot procedure was previously described [51]. Finally, the fluorescence signal was determined by super Western blot ECL substrates (PUMOKE, Wuhan, China). Detailed information of the primary and secondary antibodies were as follows: Kv1.3 (1:200, Alomone Labs, Jerusalem, Israel), CD68 (1:1000, Proteintech, Wuhan, China), iNOS (1:1000, Cell Signaling Technology, Danvers, MA, USA), IL-1β (1:1000, Abcam, Cambridge, UK), IL-6 (1:1000, Proteintech, Wuhan, China), TNF-α (1:1000, Proteintech, Wuhan, China), TRAF6 (1:1000, Proteintech, Wuhan, China), p-IKKβ (1:1000, Cell Signaling Technology, Danvers, MA, USA), IKKβ (1:1000, Cell Signaling Technology, Danvers, MA, USA), NF-κB p65 (1:1000, Cell Signaling Technology, Danvers, MA, USA), NF-κB phosphorylated p65 (p-p65; 1:1000, Cell Signaling Technology, Danvers, MA, USA), and corresponding HRP-conjugated secondary antibody (1:10,000, Proteintech, Wuhan, China). Protein expression was presented relative to the level of GAPDH (1:10,000, Proteintech, Wuhan, China).

### 4.10. Flow Cytometry

Following stimulation, microglia were double-washed with PBS. Then, cells were incubated in the Kv1.3 antibody (1:100, Alomone Labs, Jerusalem, Israel) for 2 h at 37 °C and goat anti-guinea pig IgG FITC (1:100, Bioss, Beijing, China) for 1 h at 37 °C. Then, any unbound antibody was double-washed with PBS, and the cells were suspended in 300 μL PBS and analyzed by flow cytometer (Beckman Coulter, Miami, FL, USA).

### 4.11. ELISA

The protein levels of IL-6 and TNF-α in the supernatants of microglia were quantified using ELISA kits (IL-6, 4A Biotech Co., Ltd., Beijing, China; TNF-α, Multisciences, Hangzhou, China). The microtiter plate was pre-coated with anti-mouse IL-6 and TNF-α monoclonal antibodies, and moderately diluted samples and standards were added, in which IL-6 and TNF-α would bind to their monoclonal antibodies. Next, biotinylated anti-mouse antibodies were added, which connected with mouse IL-6 and TNF-α bound to monoclonal antibodies to form an immune complex. Then, horseradish peroxidase-labeled avidin was added and bound explicitly to biotin. Finally, chromogenic reagent and stop solution were added, and the OD value was measured at 450 nm. The concentration of IL-6 and TNF-α were proportional to the OD450 value, and the concentrations of IL-6 and TNF-α were calculated by drawing a standard curve.

### 4.12. The Intracellular Concentration of Ca^2+^ Determination Assay

After treatments and being washed three times with PBS, BV2 microglia were incubated with 2 μM Fluo-4 AM fluorescent probe (Beyotime Biotechnology, Shanghai, China) for 30 min. Then, any unbound fluorescent probe was washed with PBS, and the cells were suspended in 300 μL PBS for 30 min to ensure complete intracellular conversion of Fluo-4 AM into Fluo-4. Finally, the intracellular Ca^2+^ level was quantified using flow cytometer.

### 4.13. Statistical Analysis

The data were presented as the mean ± standard error of the mean (SEM). GraphPad Prism 8.0 software was used to analyze the data and generate graphs. All of the statistical details of experiments can be found in the figure legends, including the statistical tests used, number of mice in animal experiments, and number of replicates for cell experiments. The data between two groups were analyzed using unpaired *t*-tests or unpaired *t*-tests with Welch’s correction, while one-way ANOVA with Tukey’s multiple comparisons tests were applied for more than two groups. Racine Scores of KA and KA+PAP-1 mice were analyzed using Mann–Whitney test. *p* < 0.05 was considered to suggest statistical significance.

## Figures and Tables

**Table 1:** Primer sequences.

Gene	Forward Primer (5′-3′)	Reverse Primer (5′-3′)
Kv1.1	GCATCGACAACACCACAGTC	CGGCGGCTGAGGTCACTGTCAGAGGCTAAGT
Kv1.2	GGTTGAGGCGACCTGTGAAC	TCCTCCCGAAACATCTCCATT
Kv1.3	GGAGACCTTGTGCATCATCTG	CCCATTACCTTGTCGTTCAGC
IL-1β	GAAATGCCACCTTTTGACAGTG	TGGATGCTCTCATCAGGACAG
IL-6	CATGTTCTCTGGGAAATCGTGG	GTACTCCAGGTAGCTATGGTAC
TNF-α	GGCATGGATCTCAAAGACAACC	CAGGTATATGGGCTCATACCAG
GAPDH	AGGTCGGTGTGAACGGATTTG	TGTAGACCATGTAGTTGAGGTCA

## Data Availability

Not applicable.

## Supplementary Materials

The following supporting information can be downloaded at: https://www.mdpi.com/article/10.3390/ijms232314693/s1, Figure S1: Kv1.3 expression was increased in primary microglia stimulated by LPS; Figure S2: Blockade of Kv1.3 reduced microglial activation and proinflammatory cytokine production in cultured primary microglia.
