# Supplementary material for: Blockade of Kv1.3 Potassium Channel Inhibits Microglia-Mediated Neuroinflammation in Epilepsy
Source: Int J Mol Sci. 2022 Nov 24;23(23):14693. doi: 10.3390/ijms232314693 (PMC9740890; doi:10.3390/ijms232314693)
Supplement: Supplementary file 1 [file ijms-23-14693-s001.zip › ijms-1985821-supplementary.pdf]

Supplementary Materials:

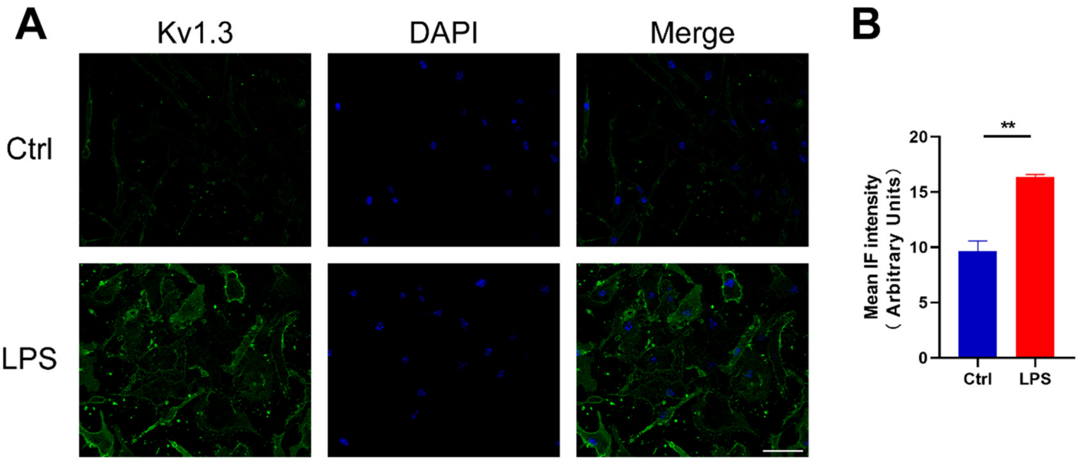

Figure S1. Kv1.3 expression was increased in primary microglia stimulated by LPS. (**A, B**) Immunofluorescence of LPS-induced Kv1.3 protein expression in primary microglia (n = 3), statistics by unpaired *t*-test. Scale bar: 20 μm. Data were depicted as the mean ± SEM. $^{**}P < 0.01$.

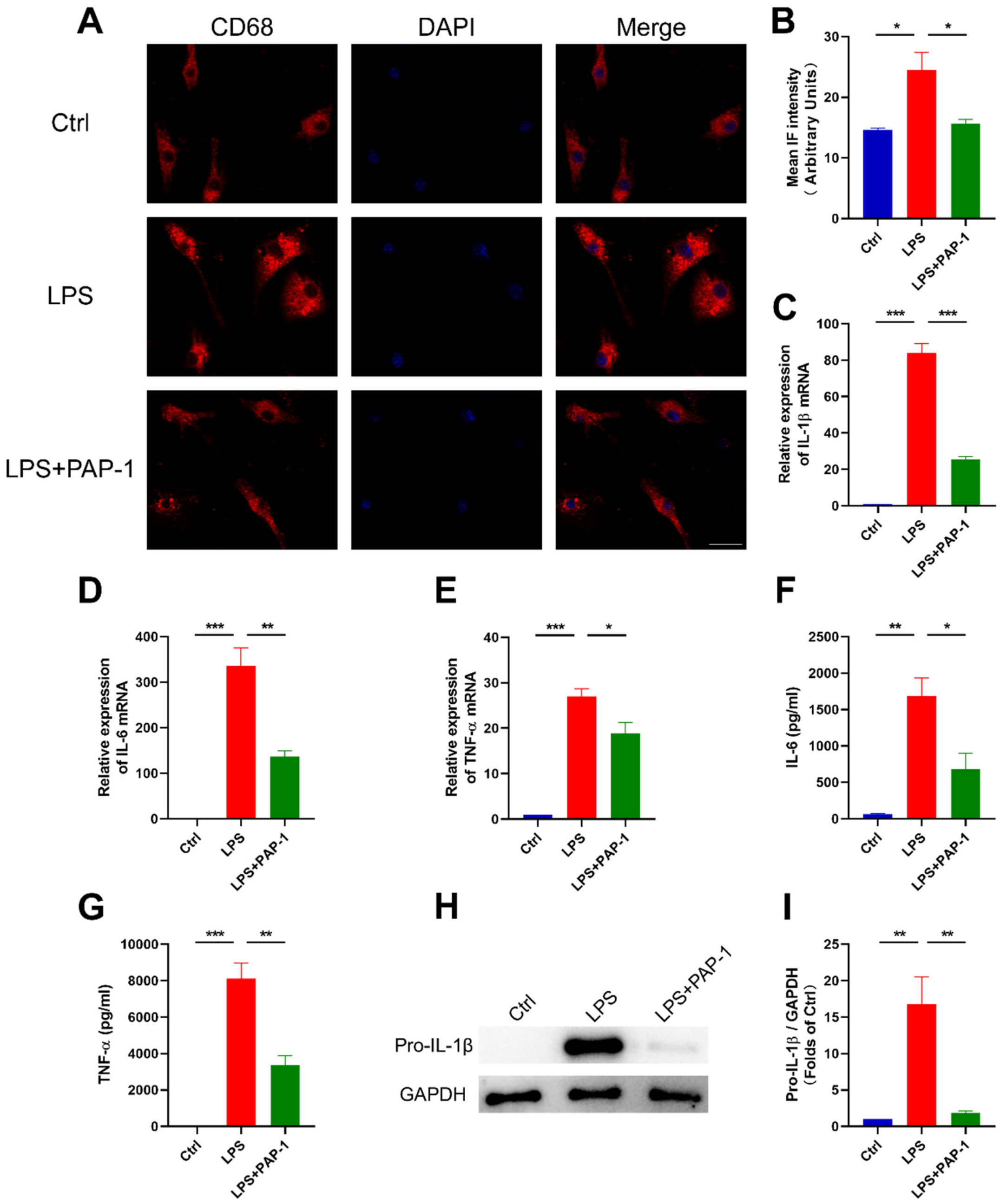

Figure S2. Blockade of Kv1.3 reduced microglial activation and proinflammatory cytokine production in cultured primary microglia. (**A**, **B**) Representative confocal images showing CD68 expression after treatment in primary microglia and statistical analysis (n = 3), statistics by one-way ANOVA with Tukey's multiple comparisons test, $F (2, 6) = 9.685$, $P = 0.0132$. Scale bar: 20 μm. (**C**-**E**) qPCR analysis of primary microglia after PAP-1 treatment showing reduced mRNA expression of proinflammatory factors (n = 3), statistics by one-way ANOVA with Tukey's multiple comparisons test; IL-1β, $F (2, 6) = 187.5$, $P < 0.0001$; IL-6, $F (2, 6) = 50.95$, $P = 0.0002$; TNF-α, $F (2, 6) = 61.66$, $P < 0.0001$. (**F**, **G**) IL-6 and TNF-α in the supernatants of primary microglia were measured using ELISA (n = 3), statistics by one-way ANOVA with Tukey's multiple comparisons test; IL-6, $F (2, 6) = 18.35$, $P = 0.0028$; TNF-α, $F (2, 6) = 48.73$, $P = 0.0002$. (**H**, **I**) Representative Western blot images and statistical analysis of IL-1β in the primary microglia (n = 3), statistics by one-way ANOVA with Tukey's multiple comparisons test, $F (2, 6) = 16.72$, $P = 0.0035$. Data were depicted as the mean ± SEM. $^{*}P < 0.05$, $^{**}P < 0.01$, $^{***}P < 0.001$.
